# Annotations

**Published:** 1907-11-16

**Authors:** 


### Annotations.

### Natural Causes of Sudden Death.

In a highly interesting paper entitled "Twelve Years' Experience of a London Coroner," Dr. W. Wynn Westcott quotes some records of the cases of sudden death from natural causes which have come under his notice. The most frequent causes of sudden death in adults are cerebral hæmorrhage, and syncope from cardiac valvular disease or fatty degeneration of the heart. Rupture of an aortic aneurysm is another fairly frequent cause of sudden death; of the deaths occurring under this head 68 per cent. were males, and of these a large proportion had been soldiers, and in many, evidences of tertiary syphilis were present. In very old people sudden syncope and passive congestion are noted as the most frequent terminations of senile decay. The influence of fracture of the neck of the femur with subsequent confinement to bed is recognised as a frequent circumstance leading to the death of elderly people. Deaths from hæmatemesis were rare, not one in a year. Hæmoptysis from phthisis pulmonalis, on the other hand, was responsible for an average of twenty deaths per annum, and of the persons so dying 75 per cent. were males. This harmonises with clinical experience, which records the comparative infrequency of severe hæmoptysis in women. Dr. Wynn Westcott notes that in many of these cases the hæmorrhage comes on quite apart from sudden strain or effort. Altogether his paper is a most valuable one, and well illustrates the service which the Medico-Legal Society may render to the general sum of medical knowledge. It is published in the last volume of the Society's Transactions.

### Diagnosis and Treatment by Correspondence.

The trial of the libel action Dakhyl *versus* Labouchere has provided another convincing demonstration of what can be done in the direction of extracting money from the suffering public by a campaign of free and unrestrained advertisement. All that is necessary is to make a series of unbounded and confident claims and to assert these without ceasing. According to the evidence submitted to the Court, the notorious Drouet Institute had a volume of correspondence which demanded the services of six or seven clerks who each wrote seventeen letters a day. From this may be plainly judged the success of the advertisements in which the virtues of this organisation were wont to be proclaimed. All this, it must be remembered, arose out of a claim on the part of the Institute to be able from written statements to diagnose the exact cause of defective hearing in any case of deafness, and to prescribe appropriate treatment. To any medical man, and one would have thought to any person of ordinary intelligence, such a claim is, on the face of it, preposterous nonsense, and ought to be condemned as humbug and quackery. Yet, doubtless on legal advice, it was deemed necessary in the above case, in order to convince a special jury of the truth of the proposition just stated, to call into the witness-box some half-dozen aural surgeons, and to register their evidence against the practice in question. Such a necessity affords room for melancholy reflection on the ease with which the general public may be humbugged in medical matters. Yet it is fair to recognise that here, as in other similar matters, the courage and resolution necessary to expose humbug and pretence were provided by a lay journal. The profession does not always see eye to eye with the editor of *Truth*, but they gladly recognise the public service he has rendered over a long course of years by his unrelenting campaign against quacks and quackery.

### The Birthday Honours.

Critics and cynics may sometimes condemn the habit of bestowing public honours and decorations as a means of recognising professional merit: yet, however logical the argument in this direction may be, it remains true that such methods of recognition have their value; and, further, they are by no means ungrateful to the profession at large. They at least have this virtue—they proclaim publicly that medical science and medical service occupy a position of high importance in the welfare of the body politic. On the present occasion there will be no lack of cordiality in the congratulations which will be offered by the profession to those of their number who have been selected for titular distinction. Professor Thos. Clifford Allbutt, who receives the K.C.B., has long occupied a conspicuous and honoured prominence among British physicians. The thoroughness and scientific character of his work attracted attention from the outset, and appropriately led in due course to his present position as Professor of Medicine in the University of Cambridge. There he has done much, both by general reputation and by efficient teaching and organisation, to build up the medical school to the influential position which it holds to-day. By his numerous writings he has contributed very largely to the formation of medical opinion, and this both in reference to scientific and to philosophic questions. It is certainly most seemly that so learned a physician, so profound a thinker, and so graceful a writer should be deemed worthy of recognition in the Honours List. Dr. Wm. H. Allchin, who receives the honour of knighthood, has held a number of important professional positions, and has taken an active interest in the reorganisation of the University of London. Lieut.-Colonel Geo. T. Beatson, C.B., is one of the surgeons to the Glasgow Western Infirmary and to the Glasgow Cancer Hospital, and is well known in the medical school of that city. As commanding officer, he has ably directed the local companies of the R.A.M.C. (Volunteers), and has brought them to a high level of success. He has also taken a great interest in the St. Andrews Ambulance Association, and he occupied a prominent part in the organisation of the Scottish Red Cross Hospital for the Boer War. For these and other services he is now promoted to be K.C.B. A knighthood has also been conferred on Dr. William Jno. Thompson, Physician in Ordinary to the Lord Lieutenant of Ireland.

